# Postoperative dental morbidity in children following dental treatment under general anesthesia

**DOI:** 10.1186/s12903-018-0545-z

**Published:** 2018-05-10

**Authors:** Yu-Hsuan Hu, Aileen Tsai, Li-Wei Ou-Yang, Li-Chuan Chuang, Pei-Ching Chang

**Affiliations:** 1 Department of Pediatric Dentistry, Chang Gung Memorial Hospital, Linkou, Taiwan, Republic of China; 2 Department of Pediatric Dentistry, Chang Gung Memorial Hospital, No. 5 Fu-Hsing Street. Kuei Shan Hsiang, Taoyuan, Taiwan, Republic of China

**Keywords:** General anesthesia, Morbidity, Pediatric dentistry

## Abstract

**Background:**

General anesthesia has been widely used in pediatric dentistry in recent years. However, there remain concerns about potential postoperative dental morbidity. The goal of this study was to identify the frequency of postoperative dental morbidity and factors associated with such morbidity in children.

**Methods:**

From March 2012 to February 2013, physically and mentally healthy children receiving dental treatment under general anesthesia at the Department of Pediatric Dentistry of the Chang Gung Memorial Hospital in Taiwan were recruited. This was a prospective and observational study with different time evaluations based on structured questionnaires and interviews. Information on the patient demographics, anesthesia and dental treatment performed, and postoperative dental morbidity was collected and analyzed. Correlations between the study variables and postoperative morbidity were analyzed based on the Pearson’s chi-square test. Correlations between the study variables and the scale of postoperative dental pain were analyzed using the Mann-Whitney U test.

**Results:**

Fifty-six pediatric patients participated in this study, with an average age of 3.34 ± 1.66 years (ranging from 1 to 8 years). Eighty-two percent of study participants reported postoperative dental pain, and 23% experienced postoperative dental bleeding. Both dental pain and bleeding subsided 3 days after the surgery. Dental pain was significantly associated with the total number of teeth treated, while dental bleeding, with the presence of teeth extracted. Patients’ gender, age, preoperative dental pain, ASA classification, anesthesia time, and duration of the operation were not associated with postoperative dental morbidity.

**Conclusion:**

Dental pain was a more common postoperative dental morbidity than bleeding. The periods when parents reported more pain in their children were the day of the operation (immediately after the procedure) followed by 1 day and 3 days after the treatment.

## Background

In developing countries, dental caries remain one of the most prevalent health problems in children. In most cases, dental treatment can be completed once children’s behaviors are properly managed. However, for very young children, medically compromised children, and those who suffer extreme anxiety, mental or physical disabilities, general anesthesia (GA) will be needed [1].

The public’s perception of GA has evolved in recent years and the use of GA has become more widely accepted [2–4]. The advantages rendered by GA include safety, efficiency, convenience, and high-quality restorative and preventive dental care [2]. Dental treatment under GA can also be completed during one single visit and minimize distress to the patient, parent, and dentist. However, children undergoing dental rehabilitation under GA do commonly experience postoperative symptoms such as dental pain and bleeding. However, dental practitioners usually have limited contact with patients after such treatment. Some studies revealed mild-to-moderate dental pain (16 to 48%) after dental treatment under GA [5–7]. Other studies found severe dental pain (74 to 95%) was the most common complication [8, 9]. While some studies have shown the prevalence of postoperative dental morbidity to be significant [8, 10–12], others have found it to be minimal [5, 13]. It is difficult to compare these studies because of the different variables used, such as the medical and cognitive status of subjects, socioeconomic status of caregivers, pain scales, standardization of GA and dental procedures, the number and types of dental procedures and postoperative analgesic usage.

**Fig. 1:**
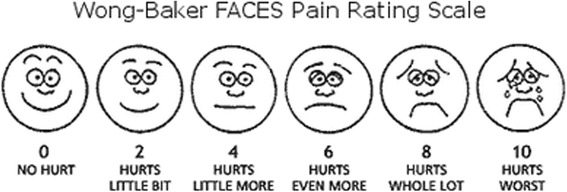
Wong-Baker FACES Pain Rating Scale

Pain is a subjective phenomenon that varies from person to person, and the gold standard for pain assessment is self-reported pain [14, 15]. In the present study, pain was assessed using the Wong-Baker FACES Pain Rating Scale (Fig. 1), a self-report rating system that is easy to use for assessing the intensity of children’s pain [15]. The scale shows a series of faces ranging from a happy face at 0 which represents “no hurt” to a crying face at 10 which represents “hurts worst.” This scale has been validated for pediatric patients between 2 and 12 years of age as well as parents who report the pain intensity on their child’s behalf [16].

However, the majority of the studies were conducted among patient populations in Western countries. Our study reported data collected among Taiwanese patient populations with the goal of identifying (1) the frequency and duration of postoperative dental morbidity of dental pain and dental bleeding, and (2) the impact of selected variables (patient demographics, intraoperative data, and types of dental treatment) on postoperative dental morbidity in children.

## Methods

This was an one-year prospective, descriptive, and comparative study among physically and mentally healthy children. All physically and mentally healthy children who were scheduled for dental treatment under GA at the Department of Pediatric Dentistry of the Chang Gung Memorial Hospital in Taiwan were included in our study. Physically and mentally compromised children were excluded from the study. A total of 56 children participated in the study from March 2012 to February 2013. Approval from the Institutional Review Board (100-2964B) for the study was also obtained by the Ethical Committee of the Chang Gung Memorial Hospital, Taoyuan, Taiwan. The study was explained to all participants in detail, including both the principle and germane risks involved. Written informed consent was obtained from participants’ legal guardians in accordance with the ethical principles of the World Medical Association agreed upon in the Declaration of Helsinki (version 2002).

After patients arrived in the operating room, standard anesthetic procedures were applied by the anesthesiologist. Nasotracheal intubation was performed on all children. The induction and maintenance agents used were sevoflurane. Dental treatment was performed by four pediatric dentists in accordance with the Guidelines of the American Academy of Pediatric Dentistry [17], including composite resin restoration, pulp treatment, stainless steel crowns (SSCs) of posterior teeth, strip crowns (SCs) of anterior teeth, and extraction of carious teeth or supernumerary teeth. After the dental treatment, children were moved to the post-anesthesia recovery room and later discharged on the same day. No children took postoperative analgesic medications such as acetaminophen.

Preoperative, intraoperative, and postoperative data were recorded. Preoperative data included gender, age, chief complaint, and preoperative dental pain (if any). Intraoperative data included the American Society of Anesthesiologist (ASA) Classification, method of intubation, induction and maintenance agents used, the number of teeth treated, and the type of treatment. Postoperative dental morbidity data were collected through questionnaires in the post-anesthesia recovery room the day of the operation (usually 1 h postoperatively) as well as 1 day, 3 days, 7 days and 14 days postoperatively. Dental pain and dental bleeding were the postoperative dental morbidity that the current study focused on. The questionnaires covered the following items: (1) Did the children have dental pain? and (2) Did the children have dental bleeding? Due to the young age of the children in the study (ranging from 1 to 8 years), the parents were the ones that reported children’s pain intensity using the Wong-Baker FACES Pain Rating Scale. Before the dental treatment, parents were instructed on how to rate their children’s postoperative pain. Postoperative dental pain was labeled as “yes” if the rating reported was equal to or greater than 2.

Statistical analysis of the data collected was performed using SPSS version 16.0 (SPSS, Inc., Chicago IL, USA). Descriptive data included the duration and frequency of postoperative morbidity. Correlations between the study variables and postoperative morbidity were analyzed based on the Pearson’s chi-square test. Correlations between the study variables and the scale of postoperative dental pain were analyzed using the Mann-Whitney U test. A *p*-value less than 0.05 was considered statistically significant.

## Results

**Fig. 2:**
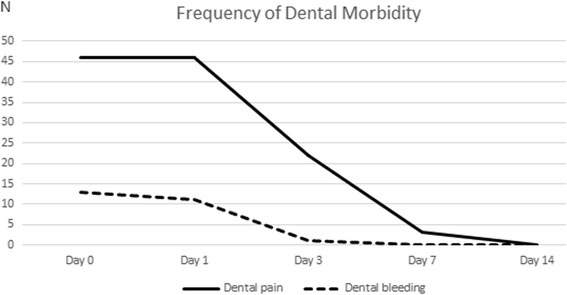
Frequency of postoperative dental pain and dental bleeding

The demographics and intraoperative data of the children are presented in Table 1. The mean age of the children was 3.34 ± 1.66 years, ranging from 1 to 8 years. Fifty-one children (91%) were brought by parents for caries treatment, and five (9%), for extraction of supernumerary teeth. The mean anesthesia time was 208.89 ± 67.27 min, and the mean duration of operation was 192.70 ± 67.40 min. The type distribution of the teeth treated is reported in Table 2, and the frequency of postoperative dental pain and bleeding, in Table 3 and Fig. 2.

**Table 1:** Demographics and intraoperative data of the children

Gender			Age (Year)		ASA Classification		Anesthesia Time (Hour)		Duration of Operation (Hour)	
Variable	Male	Female	< 3	≧3	I	II	< 3	≧3	< 3	≧3
Number (%)	31 (55)	25 (45)	20 (36)	36 (64)	18 (32)	38 (68)	18 (32)	38 (68)	22 (39)	34 (61)

**Table 2:** Type distribution of the teeth treated

	Total number of teeth treated		Teeth restored		Pulp treatment		Teeth extracted		SSC		SC	
Mean ± S.D.	13.79 ± 4.86.		7.16 ± 3.81		6.54 ± 4.23		0.64 ± 1.39		3.48 ± 2.77		2.32 ± 1.97	
Number of teeth	< 14	≧14	< 7	≧7	< 7	≧7	0	≧1	< 3	≧3	< 2	≧2
Number of children (%)	22 (39)	34 (61)	26 (46)	30 (54)	29 (52)	27 (48)	41 (73)	15 (27)	21 (38)	35 (62)	21 (38)	35 (62)

*SC* Strip Crown, *S.D.* Standard Deviations, *SSC* Stainless Steel Crown

**Table 3:** Frequency of postoperative dental pain and bleeding

	Day 0	Day 1	Day 3	Day 7	Day 14
Dental pain N (%)	46 (82)	46 (82)	22 (39)	3 (5)	0 (0)
Dental bleeding N (%)	13 (23)	11 (20)	1 (2)	0 (0)	0 (0)

The associations between postoperative dental morbidity and children’s gender, age, ASA classification, anesthesia time, duration of the operation, and the type of treatment are presented in Table 4. Postoperative dental pain did not vary with children’s gender, age, ASA classification, anesthesia time, and duration of the operation in a statistically significant way. Postoperative dental pain did, however, vary significantly with the total number of teeth treated. A significantly higher frequency of dental pain was associated with the group where the total number of teeth treated was equal to or greater than 14, compared with the group where the total number was less than 14 (*p* < 0.05). Meanwhile, postoperative dental pain was not significantly related to the number of teeth restored with composite resin, the number of teeth with stainless steel crowns, the number of teeth with strip crowns, the number of pulp treatment received, or the number of teeth extracted, respectively.

**Table 4:** Associations between postoperative dental morbidity and study variables

		Dental pain (*N* = 50)	Dental bleeding (*N* = 15)
Gender	Male	27(54)	10(66.7)
	Female	23(46)	5(33.3)
Age (Year)	< 3	18(36)	4(26.7)
	≧3	32(64)	11(73.3)
ASA Classification	I	17(34)	4(26.7)
	II	33(66)	11(73.3)
Anesthesia time (Hour)	< 3	14(28)	4(26.7)
	≧3	36(72)	11(73.3)
Duration of operation (Hour)	< 3	18(36)	5(33.3)
	≧3	32(64)	10(66.7)
Total number of teeth treated	< 14	17(34)	5(33.3)
	≧14	33(66)*	10(66.7)
Number of teeth restored	< 7	23(46)	8(53.3)
	≧7	27(54)	7(46.7)
Number of pulp treatment	< 7	24(48)	6(40)
	≧7	26(52)	9(60)
Number of teeth extracted	0	37(74)	8(53.3)
	≧1	13(26)	7(46.7)*
Number of teeth with SSC	< 3	17(34)	4(26.7)
	≧3	33(66)	11(73.3)
Number of teeth with SC	< 2	18(36)	7(46.7)
	≧2	32(64)	8(53.3)

*p < 0.05 (Pearson’s chi-square test)

The scale of postoperative dental pain as related to the total number of teeth treated was further analyzed and presented in Table 5. The highest pain rating was recorded in the post-anesthesia recovery room (1 h postoperatively). The ratings gradually dropped over 1 day, 3 days, 7 days, and 14 days postoperatively. The scale of postoperative dental pain was significantly related to the total number of teeth treated, in the recovery room as well as 1 day and 3 days postoperatively. This significance disappeared as more days passed. Although higher postoperative pain ratings during the first 3 days were seen with the group experiencing preoperative dental pain, this difference was not significant between the group that experienced preoperative dental pain and the group that did not.

**Table 5:** Mean ratings of postoperative dental pain related to preoperative pain and the total number of teeth treated

	Preoperative dental pain			Total number of teeth treated		
	No (*N* = 31)	Yes (*N* = 25)	*p* value	< 14 (*N* = 22)	≧14 (*N* = 34)	*p* value
Day 0	3.03 ± 2.36	4.08 ± 2.80	0.180	2.82 ± 2.52	3.94 ± 2.58	0.049*
Day 1	2.52 ± 1.93	3.28 ± 1.62	0.122	1.91 ± 1.80	3.47 ± 1.58	0.002*
Day 3	0.77 ± 1.33	1.20 ± 1.30	0.135	0.55 ± 1.10	1.24 ± 1.39	0.045*
Day 7	0.13 ± 0.50	0.08 ± 0.40	0.688	0.00 ± 0.00	0.18 ± 0.58	0.156
Day 14	0.00 ± 0.00	0.00 ± 0.00	1.000	0.00 ± 0.00	0.00 ± 0.00	1.000

*p < 0.05 (Mann-Whitney U test)

The rating of postoperative dental pain as related to the dental procedure performed was further analyzed and presented in Table 6. The highest pain rating was associated with exodontia, followed by restoration with SSCs. The postoperative dental pain rating was significantly related to the number of teeth receiving pulp therapy 1 day postoperatively. The postoperative dental pain rating was also significantly related to the number of teeth restored with SSCs, in the recovery room as well as 1 day and 3 days postoperatively.

**Table 6:** Mean ratings of postoperative dental pain related to dental procedures

	Number of teeth restored with composite resin		Number of teeth treated with pulp therapy		Number of teeth extracted		Number of teeth restored with SSC		Number of teeth restored with SC	
	< 7 (*N* = 26)	≧7 (*N* = 30)	< 7 (*N* = 28)	≧7 (N = 28)	0 (*N* = 41)	≧1 (N = 15)	< 3 (*N* = 21)	≧3 (*N* = 35)	< 2 (N = 22)	≧2 (N = 34)
Day 0	3.15 ± 2.48	3.67 ± 2.78	3.07 ± 2.34	3.93 ± 2.80	3.12 ± 2.15	4.53 ± 3.42	2.38 ± 1.96*	4.17 ± 2.72*	3.18 ± 3.20	3.06 ± 2.04
Day 1	2.69 ± 1.78	3.00 ± 1.88	2.21 ± 1.75*	3.50 ± 1.69*	2.63 ± 1.76	3.47 ± 1.92	1.81 ± 1.54*	3.49 ± 1.70*	2.91 ± 1.93	2.82 ± 1.78
Day 3	0.77 ± 1.27	1.13 ± 1.36	0.64 ± 1.10	1.29 ± 1.46	0.83 ± 1.18	1.33 ± 1.63	0.38 ± 0.80*	1.31 ± 1.45*	1.00 ± 1.35	0.94 ± 1.32
Day 7	0.08 ± 0.39	1.13 ± 0.51	0.07 ± 0.38	0.14 ± 0.52	0.10 ± 0.44	0.13 ± 0.52	0.10 ± 0.44	0.11 ± 0.47	0.00 ± 0.00	0.18 ± 0.58
Day 14	0.00 ± 0.00	0.00 ± 0.00	0.00 ± 0.00	0.00 ± 0.00	0.00 ± 0.00	0.00 ± 0.00	0.00 ± 0.00	0.00 ± 0.00	0.00 ± 0.00	0.00 ± 0.00

*p < 0.05 (Mann-Whitney U test)

Postoperative dental bleeding did not vary significantly with children’s gender, age, ASA classification, anesthesia time, and duration of the operation. The presence of teeth extracted was significantly related to postoperative dental bleeding. A significantly higher frequency of dental bleeding was found in the children with teeth extracted, compared with the children without teeth extracted (*p* < 0.05). Postoperative dental bleeding was not significantly related to the total number of teeth treated, the number of teeth restored (with composite resin, stainless steel crown, or strip crown), or the number of pulp treatment received.

## Discussion

All physically and mentally healthy children receiving various dental treatments under general anesthesia in this study saw their treatment completed in one single visit to the Department of Pediatric Dentistry. The treatments performed included composite resin restoration, pulp therapy, stainless steel crowns (SSCs) of posterior teeth, strip crowns (SCs) of anterior teeth, and extraction of carious teeth or supernumerary teeth. Postoperative morbidity of dental pain and bleeding was observed. Postoperative morbidity of dental pain and bleeding was observed in 46 and 13 children respectively among the 56 children evaluated in this study.

Earlier studies have found that the most common postoperative dental complication is toothache [8, 9, 16] and that the incidence of postoperative pain after dental rehabilitation under GA ranges from 36 to 93% [8, 10–12, 18]. Children in the present study thus appeared to experience a higher rate of dental pain (82%), which may be attributable to the higher number of dental procedures performed and longer duration of treatment. In addition, the induction and maintenance agent of anesthesia used in our study, sevoflurane, has been associated with more pain than halothane as reported by Ersin et al. [18].

In terms of the postoperative pain associated with particular dental treatments, the pain ratings were significantly related to pulp therapy and restoration with SSCs for teeth with pulp infection or inflammation. Gingivitis caused by poor oral hygiene among children receiving SSCs also induced more pain sensation after the treatment. On the other hand, a lower percentage (26%) of the children receiving tooth extraction experienced postoperative dental pain, which may be attributable to the local anesthesia administered before extraction. This echoes the finding by Atan et al. that the frequency of postoperative dental pain was lower in children who received local anesthesia [8]. Hence, when dental extractions are performed under general anesthesia, it is important to ensure that appropriate pain medication is provided.

Postoperative dental pain was found to correlate with the total number of teeth treated in our study. Children with 14 or more teeth treated experienced a significantly higher frequency of dental pain (*p* < 0.05) than those with a lower number of teeth treated. The current study also found that 82% of the children experienced dental pain both 1 h and 1 day after the operation, and 39% continued experiencing pain 3 days after the operation. Seven days after the operation, dental pain lingered among 5% of the children before completely stopping 14 days after the operation. These results are consistent with the findings reported by Needleman et al. and Atan et al. that postoperative dental pain occurred within 24 h and 1 day after the operation before subsiding 1 week after the operation [8, 9].

In this study, no pain killer or other medication for pain control was administrated to children. Since the highest pain rating (3.50 ± 2.59) was recorded in the postoperative recovery room, analgesics could be administered intravenously approximately 30 min before the termination of general anesthesia to reduce the pain that may occur during the first hour postoperatively. The higher pain rating was also associated with a higher number of teeth treated 1 day and 3 days postoperatively. As the benefit of administering anti-inflammatory analgesics to control toothache in patients treated under GA has been documented [4, 5, 19], it is important to instruct parents to give analgesics regularly for the first few days postoperatively instead of waiting until the pain occurs [9]. According to the results of our study, in the case of comprehensive dental treatment, analgesic medication is recommended for at least 3 days postoperatively to prevent postoperative pain or lower the frequency and degree of the pain.

Postoperative bleeding was less frequent than postoperative dental pain in our study. Twenty-three per cent of the children experienced bleeding 1 h postoperatively, 20% experienced bleeding 1 day after the operation, and 3% experienced bleeding 3 days after the operation. No bleeding was found 7 and 14 days after the operation. The results of our study are consistent with the findings of Mayeda et al. that postoperative dental bleeding subsided within 24 h after the operation [7].

Given most Taiwanese parents’ reticence toward tooth extraction, we endeavored to preserve the tooth treated even under general anesthesia in this study, which resulted in a lower extraction rate and in turn a lower rate of postoperative bleeding. The presence of extracted teeth was found to correlate with postoperative bleeding, as evidenced in the significantly higher frequency of dental bleeding (*p* < 0.05) in the group with teeth extracted. Blood clots formed over the extraction wound 1 day after the extraction, followed by epithelialization 3 to 7 days after the extraction. Bleeding usually occurred within 3 days after the extraction. There are different ways to control bleeding, such as using gauze packing, suture, and hemostasis agent. Using local anesthetic agents together with epinephrine can also reduce the incidence of bleeding during the early postoperative period. Well-controlled postoperative bleeding will reduce patients’ and parents’ anxiety.

Our study reports preliminary findings of postoperative dental morbidity after dental treatment under GA in Taiwan based on a prospective study conducted among a small sample of children. The key contribution of the study resides in the determination of the type and frequency of postoperative dental complaints among children. Future studies could exclude children under 2 or 3 and compare children’s self-reported pain intensity with that based on parents’ reporting. Given the high percentage of children who reported pain in our study, any correlation between the occurrence of pain and bleeding could be another potential topic for future studies. As the study participants were recruited only from the Taoyuan City, future studies could recruit children from other parts of Taiwan who receive dental treatment under GA. Furthermore, dental pain and bleeding were the two types of dental morbidity which the current study focused on. Similar studies could be initiated in the future among children receiving dental treatment under GA in other hospitals and with an expanded scope to include other types of postoperative morbidity.

Oral rehabilitation under general anesthesia can improve children’s oral health as well as the quality of their physical, emotional, and social life [20]. However, to address parents’ concern about the safety and postoperative morbidity related to general anesthesia, dentists should inform parents of the postoperative symptoms that may occur immediately and days after the operation under general anesthesia. Every effort must also be exerted to minimize the morbidity and ensure that both parents and children are comfortable with the procedures.

## Conclusion

Our study showed that dental pain was a more common postoperative morbidity after dental treatment under general anesthesia than bleeding. Dental pain was related to the total number of teeth treated. The periods when parents reported more pain in their children were the day of operation (immediately after the procedure) followed by 1 day and 3 days after the treatment.

## Abbreviations

ASA: American Society of Anesthesiologist
GA: General anesthesia
S.D.: Standard deviations
SC: Strip crown
SSC: Stainless steel crown
